# Chronic Lymphocytic Leukemia/Small Lymphocytic Lymphoma Presenting as Chronic Diarrhea; An Unusual Presentation of a Common Lymphoproliferative Disorder

**DOI:** 10.1155/2021/8884820

**Published:** 2021-04-26

**Authors:** Smitha Narayana Gowda, Hafez Mohammad Abdullah, Rakshya Sharma, Mohamed A. Abdallah

**Affiliations:** ^1^Department of Internal Medicine, University of South Dakota Sanford School of Medicine, Sioux Falls, SD, USA; ^2^Department of Pulmonary Medicine, University of South Dakota Sanford School of Medicine, Sioux Falls, SD, USA

## Abstract

Chronic lymphocytic leukemia (CLL) is the most common lymphoproliferative disorder in the United States. It has a variable presentation with most patients having asymptomatic lymphocytosis. Many other patients present with lymphadenopathy or enlargement of other organs of the reticuloendothelial system. However, CLL can present with extramedullary involvement. Most commonly, this is in the form of skin or central nervous system involvement, though rarely it can present with gastrointestinal involvement. Here, we present the case of a 70-year-old Caucasian male who presented with chronic diarrhea for over 4 months. After failing conservative treatment, a colonoscopy was performed which showed diffuse mucosal nodularities with a biopsy revealing CLL. The patient was treated successfully with chemotherapy and his diarrhea improved. This is a differential to keep in mind in patients with chronic diarrhea, once the more common causes have been ruled out.

## 1. Introduction

B-cell chronic lymphocytic leukemia (CLL) is the most common form of leukemia in the United States, accounting for almost 25 to 30% of all leukemias [1]. CLL is a progressive accumulation of functionally inert lymphocytes in the blood, lymphoid tissues, and bone marrow. It most commonly affects elderly patients with a median age of diagnosis being 70 years of age [2]. The presentation of CLL can be very variable ranging from asymptomatic lymphocytosis to painless lymphadenopathy, hepatomegaly, splenomegaly, cytopenias, and infections. Patients can also sometimes present with typical B-symptoms such as fever, night sweats, and unintentional weight loss. Sometimes, CLL can transform into a more aggressive form of large B-cell lymphoma called Richter's transformation which can occur in up to 10% of CLL patients and is associated with poor prognosis.

Extramedullary involvement of CLL is rare, but if it occurs, it most commonly involves the skin and central nervous system [3]. While gastrointestinal involvement can occur rarely in patients with CLL, it mostly occurs in patients with Richter's transformation and is very unusual to occur in patients with CLL [4].

## 2. Case Presentation

A 70-year-old Caucasian male presented with complaints of diarrhea for the last 4 months. He had associated fecal urgency and incontinence at times. He was having 4-5 bowel movements a day ranging from semisolid to watery stools. He denied any history of fevers, abdominal pain, vomiting, weight loss, night sweats, or skin rashes. He denied any recent travel history or exposure to anyone with similar symptoms.

On physical examination, his abdomen was soft and nontender. There was no apparent hepatomegaly or splenomegaly. The rest of the cardiovascular, respiratory, and nervous system examinations were unremarkable. Initial laboratory evaluation showed a hemoglobin of 15.5 g/dL (14–18), WBC of 8.3 K/uL (4.5–11), leucocyte fraction of 45.7% (15–50%), platelet count of 187 K/uL (140–440), creatinine of 0.9 mg/dL (0.7–1.3), AST of 34 U/L (13–39), ALT of 25 U/L (4–33), ALP of 102 U/L (34–104), albumin of 4 g/dL (3.5–5.7), and globulin of 3.1 g/dL (1.5–4.5).

**Figure 1:**
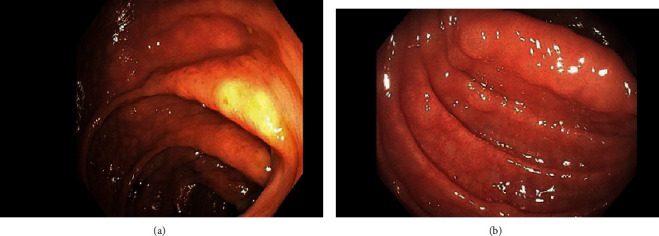
Colonoscopy showing submucosal nodularity.

**Figure 2:**
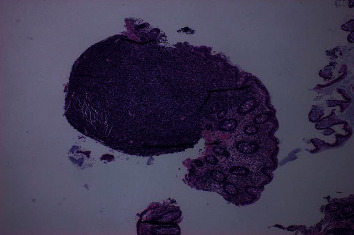
Hematoxylin and eosin stain showing lymphoid aggregate with normal colon mucosa.

**Figure 3:**
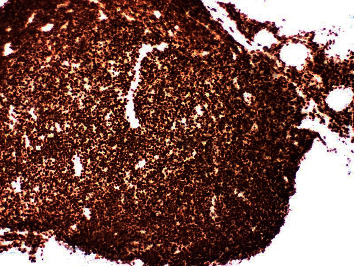
Lymphoid aggregate positive for PAX5 stain.

**Figure 4:**
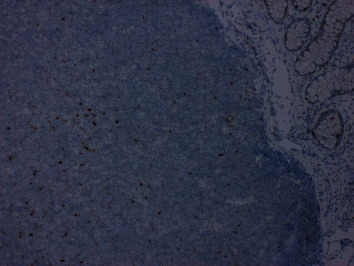
Lymphoid aggregate negative for cyclin D1.

Further workup was done for finding the etiology of the chronic diarrhea. Work up for infection, including parasitic infection and bacterial infections including *Clostridium difficile*, was negative. He underwent colonoscopy for further evaluation. This revealed a normal mucosa but diffuse submucosal nodularity through the large intestine (Figure 1). Biopsies were taken from multiple sites, and histopathology of the biopsied revealed multiple aggregates for small mature lymphocytes in the submucosa (Figure 2). There was no large cell transformation. Histopathology was negative for CMV colitis. Immunohistochemical stains revealed neoplastic lymphocytes positive for PAX5 (Figure 3), BCL-2, dim BCL-6, CD5, partial CD20, and CD23 and negative for CD3, cyclin D1 (Figure 4), and CD10. A diagnosis of chronic lymphocytic leukemia/small lymphocytic lymphoma was made.

The patient then underwent CT chest, abdomen, and pelvis with IV contrast for evaluation which revealed extensive bilateral axillary, hilar, abdominal, and pelvic lymphadenopathy. He subsequently underwent bone marrow biopsy histopathology which revealed hypercellular bone marrow, with 50% involved by lymphoid cells; flow cytometric immunophenotyping studies revealed monoclonal B-cell lymphoid population with kappa light chain restriction with CD5 and CD23 coexpression consistent with CLL. The patient did not have any abnormal cell clone harboring deletion of 11q, 13q, or 17p. His Rai staging was Stage I and Binet staging was Stage B, putting him at intermediate risk. The patient was referred to oncology that started him on obinutuzumab and venetoclax chemotherapy for the CLL.

The patient went into remission from his CLL on treatment with chemotherapy. The patient improved significantly, and the diarrhea resolved. On 3- and 6-month follow-ups, the patient experienced significant symptomatic improvement with resolution of chronic diarrhea.

## 3. Discussion

A review of the literature revealed a total of *n* = 5 reported cases including ours, where the initial presentation of CLL/SLL was chronic diarrhea [5–8]. This is presented in Table 1. While GI manifestation can occur in CLL, especially in advanced stages or during blast transformation, diarrhea as the initial presentation is rare. In the existing case reports with similar presentation, the mean age of presentation ranged from 65 to 81 years. Associated symptoms were fecal urgency, abdominal pain, and weight loss. One patient presented with abdominal pain and hematochezia and was found later to have intussusception. The duration of symptoms ranged from 4 weeks to 6 months. Three patients had no history of CLL, and one patient had history of CLL that was treated and was thought to be in remission.

Endoscopic evaluation with colonoscopy was notable for variable findings ranging from polypoid mass, multiple linear ulcerations, and ulcerations with mucosal inflammation and friability. Imaging with contrast enhanced CT in all cases revealed abdominal and pelvic lymphadenopathy. Colonic histopathology in all cases revealed lymphocytic infiltration. Treatment details were available for two patients; polypectomy was done for one patient, and rituximab was used to treat for the other patient with resultant resolution of symptoms. In the remaining two patients, one patient decided to pursue comfort care measures and passed away shortly after hospital discharge. No treatment details were available for the other patient who also passed away few weeks after diagnosis.

Of these cases, there are two cases of CCL involving the large intestine including our patient [8]. A case of 66-year-old male who came in whose initial presentation of CLL was abdominal pain and hematochezia and was found to have intussusception on abdominal ultrasound. On further evaluation with colonoscopy, the patient was found to have intraluminal mass in the hepatic flexure. Our patient on the other hand presented with diarrhea and colonoscopy revealed submucosal nodularity in the large intestine. Histopathology of both patients revealed lymphocytic infiltrates. The patient who presented with the colonic mass died within days of diagnosis [7]. Our patient received chemotherapy and is currently in remission.

## Figures and Tables

**Table 1:** All the prior reported cases of CLL presenting with chronic diarrhea.

Case	Age	Sex	Initial presentation	History of CLL	Colonoscopy	Histopathology	Imaging	Treatment	Outcome
1	75 years [5]	M	Watery diarrhea, abdominal pain for 4 months	CLL 4 years after treatment-remission	4 cm polypoidal mass, located at the area of the cecum	B-cell small lymphocytic lymphoma	CT abdomen and pelvis revealed multiple lymphadenopathies in the abdominal and pelvic region	Polypectomy	Resolution of diarrhea after polypectomy. Outpatient follow-up

2	65 years [6]	M	Worsening diarrhea, fecal urgency for 6 months	No hx of CLL	An ulcer measuring 2 cm in diameter was noted at the cecum/ileocecal valve junction and multiple nonbleeding, superficial, linear ulcers were observed in the rectal mucosa	Dense, atypical, lymphoid infiltrate suggestive of a low-grade lymphoproliferative disorder	CT imaging showed mild splenomegaly and enlarged portacaval, precaval, periaortic, mesenteric, bilateral supraclavicular, axillary, and inguinal lymph nodes	Weekly rituximab	Resolution of diarrhea after 4 weeks of treatment

3	81 years [7]	M	Diarrhea for 6 weeks and 10-pound weight loss	No hx of CLL	Moderate mucosal inflammation, friability, and a few ulcerations, more pronounced in the sigmoid colon	Lamina propria expanded by small lymphocytes	Inflammation and colonic wall thickening and possible narrowing of the rectosigmoid colon with surrounding fat stranding and significant retroperitoneal and mesenteric lymphadenopathy	Palliative care, trial of chlorambucil	Died after 10 days at home

4	66 years [8]	M	Hematochezia, intermittent left lower abdominal pain of 1-month duration	No hx of CLL	Huge, round, reddish intraluminal mass covered with blood clots in the ascending colon just proximal to hepatic flexure that occupied nearly the entire colonic lumen	Dense cellular infiltrate in the mucosa. The infiltrating cells were darkly stained, uniformly small and round lymphocytes that had infiltrated the lamina propria but spared the crypts	Initial ultrasound: right midabdomen that had alternating hypoechoic and hyperechoic rings surrounding an echogenic center (doughnut sign), findings suggestive of intussusception	—	Died several weeks from presentation

5	70 years	M	Diarrhea for 4 months	No hx of CLL	Normal mucosa but diffuse submucosal nodularity through the large intestine	Multiple aggregates for small mature lymphocytes in the submucosa	CT chest, abdomen, and pelvis revealed extensive bilateral axillary, hilar, abdominal, and pelvic lymphadenopathy	Obinutuzumab and venetoclax chemotherapy	Asymptomatic on 6 months follow-up

## Additional Points

(1) Chronic lymphocytic leukemia (CLL) is the most common lymphoproliferative disorder in the United States. (2) It has a very variable presentation with most patients having asymptomatic lymphocytosis. (3) CLL can also present with extramedullary involvement, most commonly the skin or central nervous system. (4) Rarely, it can also present with chronic diarrhea, which typically resolves after treatment of the CLL.
